# The role of inositol 1,4,5-trisphosphate 3-kinase A in regulating emotional behavior and amygdala function

**DOI:** 10.1038/srep23757

**Published:** 2016-04-07

**Authors:** Sooyoung Chung, Il Hwan Kim, Dongmin Lee, Kyungjoon Park, Joo Yeon Kim, Yeon Kyung Lee, Eun Joo Kim, Hyun Woo Lee, June-seek Choi, Gi Hoon Son, Woong Sun, Ki Soon Shin, Hyun Kim

**Affiliations:** 1 Department of Biomedical Sciences, Brain Korea 21 PLUS, College of Medicine, Korea University, Seoul 136-705, Korea; 2 Department of Brain and Cognitive Sciences, Scranton College, Ewha Womans University, Seoul, 03760, Korea; 3 Department of Cell Biology, Duke University Medical School, Durham, North Carolina 27710, USA; 4 Department of Anatomy, College of Medicine, Korea University, Seoul 136-705, Korea; 5 Department of Biology, Department of Life and Nanopharmaceutical Sciences, Kyunghee University, Seoul 130-701, Korea; 6 Department of Psychology, Korea University, Seoul 136-701, Korea; 7 Department of Psychology, University of Washington, Seattle, WA, USA

## Abstract

Inositol 1,4,5-trisphosphate 3-kinase A (IP_3_K-A) is a molecule enriched in the brain and neurons that regulates intracellular calcium levels via signaling through the inositol trisphosphate receptor. In the present study, we found that IP_3_K-A expression is highly enriched in the central nucleus of the amygdala (CeA), which plays a pivotal role in the processing and expression of emotional phenotypes in mammals. Genetic abrogation of IP_3_K-A altered amygdala gene expression, particularly in genes involved in key intracellular signaling pathways and genes mediating fear- and anxiety-related behaviors. In agreement with the changes in amygdala gene expression profiles, IP_3_K-A knockout (KO) mice displayed more robust responses to aversive stimuli and spent less time in the open arms of the elevated plus maze, indicating high levels of innate fear and anxiety. In addition to behavioral phenotypes, decreased excitatory and inhibitory postsynaptic current and reduced c-Fos immunoreactivity in the CeA of IP_3_K-A KO mice suggest that IP_3_K-A has a profound influence on the basal activities of fear- and anxiety-mediating amygdala circuitry. In conclusion, our findings collectively demonstrate that IP_3_K-A plays an important role in regulating affective states by modulating metabotropic receptor signaling pathways and neural activity in the amygdala.

Inositol 1,4,5-trisphosphate 3-kinases (IP_3_Ks) are the most active inositol phosphate kinase detectable in mammals and play a role in the rapid metabolization of the inositol 1,4,5-trisphosphate (IP_3_) pool generated by the activation of phospholipase C (PLC)-coupled membrane receptors^1^. Rapid clearance of IP_3_ stops intracellular calcium release from the endoplasmic reticulum (ER) where IP_3_-sensitive calcium channels are located. Therefore, IP_3_Ks modulate intracellular calcium signaling induced by the activation of G-protein coupled receptors (GPCRs) associated with PLC. Mammals have three IP_3_K genes expressed in specific spatial distributions; therefore, gene expression produces different phenotypes in different cells and tissues^2^. Over-expression of IP_3_K consistently suppresses IP_3_–evoked increases in intracellular calcium in response to an agonist, whereas deletion or inactivation of different genes elicits diverse phenotypes depending on cell type. IP_3_K-A was the first IP_3_K purified and identified from rat brain and is expressed in discrete neuronal populations in mammalian forebrain structures^3^. Recent studies revealed that neuronal IP_3_K-A plays a novel role in cytoskeletal reorganization, interacting with F-actin and microtubules, which modulate neuronal plasticity^4^,^5^. For example, IP_3_K-A is enriched in dendritic spines of mature neurons and modulates actin dynamics in the hippocampus. Additionally, genetic deletion of IP_3_K-A produces deficits in long-term potentiation (LTP) in the dentate gyrus and impairs memory performance in the novel object recognition test. However, deletion did not affect spatial learning in the Morris water maze^6^,^7^.

The amygdala is required for processing and expressing emotional information, and its dysregulation is associated with emotional dysfunction^8^,^9^. The amygdala is a prime target for treating anxiety-related disorders because it couples sensory stimuli and outputs to effector regions involved in behavioral responses^10^. The amygdala contains several subnuclei with phenotypically distinct neuronal populations, each of which potentially plays a unique role in processing stress and other fear-related stimuli^8^,^11^. The basolateral nucleus of the amygdala (BLA) is highly enriched in glutamatergic principal neurons and is required for associative learning. The central nucleus of the amygdala (CeA) primarily consists of GABAergic medium spiny neurons and controls the processing and expression of emotion. The CeA constitutes the major outputs of the amygdala and mediates autonomic and behavioral correlates of fear and anxiety^12^,^13^. Growing evidence also shows that metabotropic receptor signaling mediates the cross talk and neural circuitry-dependent actions of neuropeptides and neurotransmitters that play modulatory and integrative roles in the cellular and molecular basis of emotion^14^,^15^.

Although IP_3_K-A is abundantly expressed, its role in the amygdala is still elusive. Given the findings on the IP_3_K-A knockout (KO) mice in hippocampus-dependent learning, we hypothesize that amygdala IP_3_K-A may play a role in the association between environment and emotion. Thus, we characterized molecular signatures of the amygdala in IP_3_K-A KO mice and examined the functional consequences of IP_3_K-A KO through electrophysiology and behavioral assessments.

## Results

### Amygdala expression of IP_3_K-A

**Figure 1:**
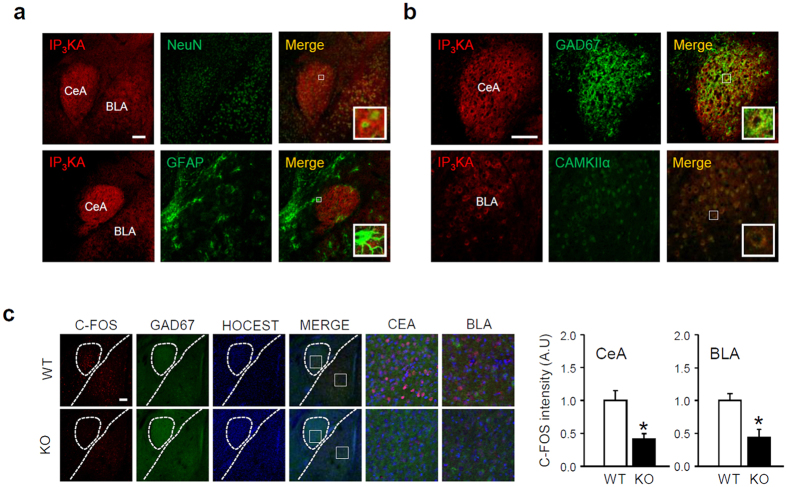
IP_3_K-A protein expression in the amygdala. (**a**) IP_3_K-A (red) immunoreactivity was examined by co-staining with markers for neurons (NeuN) or astrocytes (GFAP). All the markers are green. (**b**) IP_3_K-A positive cells were double-labeled with markers for inhibitory GABAergic neuron (GAD67) or pyramidal neuron (CAMKIIα). (**c**) C-Fos and GAD67 expression was examined in the amygdala of WT and IP_3_K-A KO mice. Relative immunoreactivity in the indicated regions are presented as mean ± SEM in arbitrary units (A.U.), in which mean densitometric intensity observed in WT mice was defined as 1 (n = 4; *p < 0.05 by *t*-test). CeA: the central nucleus of the amygdala; BLA: the basolateral nucleus of the amygdala. Scale bar: 100 μm.

In adult mice, IP_3_K-A is expressed in the forebrain, and our preliminary results indicated that the amygdala had abundant expression of IP_3_K-A gene transcripts^2^,^3^. We found that IP_3_K-A protein expression is highly enriched in the amygdala; immunoreactivity is particularly strong in the CeA and BLA (Fig. 1a). To identify cells expressing IP_3_K-A, we examined co-localization of IP_3_K-A with markers of specific cell types using immunohistochemistry. IP_3_K-A colocalized with NeuN but was barely detectable in glial fibrillary acidic protein (GFAP)-positive cells, indicating that IP_3_K-A is primarily expressed in neurons rather than glia in the amygdala (Fig. 1a). In the CeA, IP_3_K-A primarily colocalized with GAD67-positive GABAergic interneurons, whereas most IP_3_K-A in the BLA was in CAMKIIα-positive excitatory pyramidal neurons (Fig. 1b). To determine whether IP_3_K-A regulates neuronal activity, we compared amygdala c-Fos expression in WT and KO mice. C-Fos expression was reduced in the BLA and CeA of KO mice (Fig. 1c); therefore, IP_3_K-A may regulate amygdala function by affecting the excitatory and inhibitory components of the intra-amygdaloid circuits.

### Genome-wide amygdala mRNA expression in IP_3_K-A KO mice

**Figure 2:**
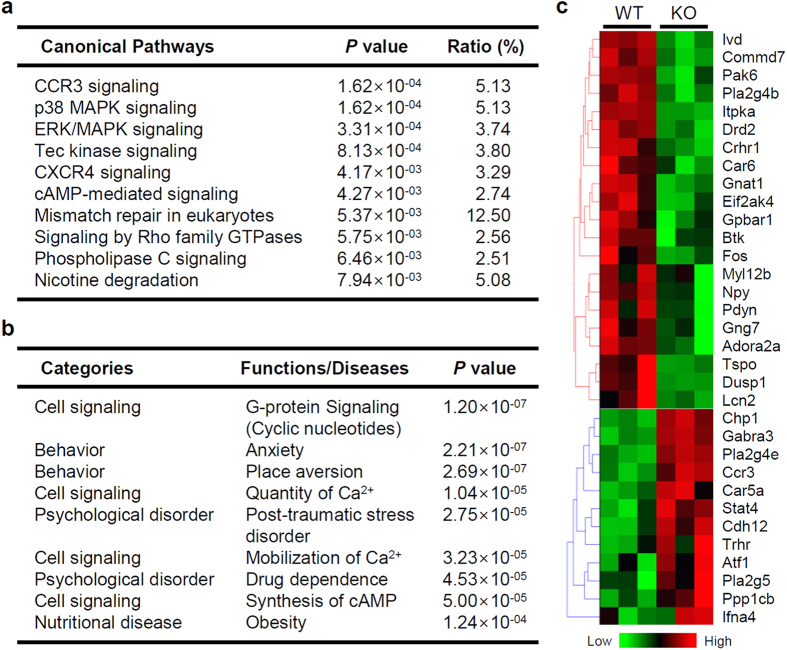
Microarray analysis on amygdala mRNA expression followed by gene set enrichment analyses. Summary of the (**a**) canonical pathways and (**b**) related biological functions and diseases predicted by the Ingenuity Pathway Analysis^TM^ software. *P* value of overlap was calculated by the Fisher’s exact test to evaluate significance of an association between our DEG set and an indicated pathway or biological function/disease. The ratio indicates the percentage of overlapping genes from the DEG set compared with the total number of genes in an indicated canonical pathway. (**c**) Heat map representation of hierarchically clustered differentially expressed genes (DEGs) related to the biological pathways/functions listed in (**a**,**b**).

Because reduced c-Fos expression is associated with changes in amygdala gene expression, we compared genome-wide mRNA expression from amygdala tissue of WT and KO mice using microarrays. We identified 265 differentially expressed gene (DEG) transcripts and categorized the DEG sets by canonical pathway and biological function/disease using Ingenuity Pathway Analysis (IPA) software. Enrichment analyses of DEGs revealed that important signaling pathways could be significantly altered by loss of IP_3_K-A, including chemokine receptor signaling, MAPK, GPCR (cyclic adenosine monophosphate [cAMP] and PLC-mediated signaling), and Rho family GTPase signaling pathways (Fig. 2a). These pathways are downstream of metabotropic receptor-initiated signals mediating various neurotransmitters, neuropeptides, and growth factors. Key signal transduction pathways (including MAPK and GPCR pathways) were biased toward inhibition (Activation Z-scores: MAPK/ERK, −2.120; MAPK/p38, −2.380; cAMP-mediated signaling, −1.633; PLC-mediated signaling, −1.516). When examining annotation of related biological function/disease, we found significant alterations in GPCR- and calcium-related signaling pathways that corresponded with the identified functions of IP_3_Ks in the canonical pathway (Fig. 2b). Importantly, a subset of genes related to anxiety-like behaviors and psychiatric illness (e.g., post-traumatic stress disorder [PTSD] and addiction) was significantly enriched in our DEG set. Representative DEGs from the analyses are summarized as a heat map presentation, including PLC- and Rho GTPase-coupled signaling and fear- and anxiety-related phenotypes (Fig. 2c).

**Figure 3:**
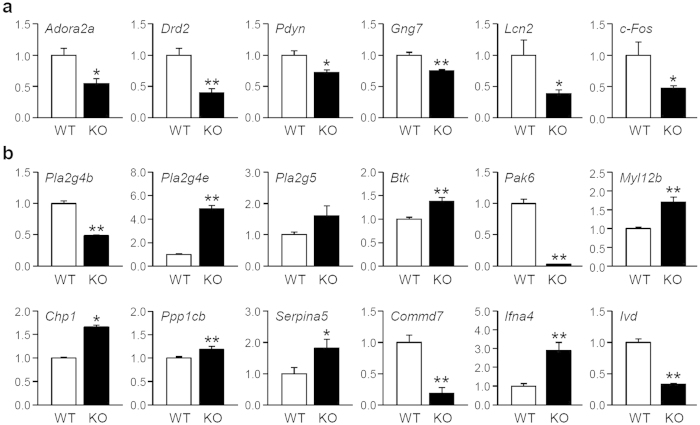
Differential gene expression profiles in the amygdala of IP_3_K-A KO mice. Quantitative RT-PCR analyses for differential expression of selected amygdala differentially expressed genes (DEGs) categorized as (**a**) fear- and anxiety-related genes and (**b**) metabotropic receptor signaling-associated genes. Relative expression levels for each mRNA were normalized by TATA box-binding protein (TBP) expression and presented as mean ± SEM in arbitrary units (A.U.), in which mean expression levels of WT mice were defined as 1 (n = 5; *p < 0.05 and **p < 0.01 by *t*-test).

Among 265 DEGs identified by IPA, we focused on eighteen DEGs chosen based on functional importance, extent of group-difference, and validated expression patterns by qRT-PCR (Fig. 3). Featured genes were in one or more functional categories listed in Fig. 2 and primarily were related to fear- and anxiety-related behaviors and/or GPCR- and calcium-associated signaling pathways. For example, genes associated with emotional abnormalities and linked with GPCR signaling (*Adora2a, Drd2, Pdyn, Gng7, Lcn2,* and *c-Fos*) were significantly down-regulated in the KO amygdala (Fig. 3a). Additionally, expression of several mRNA encoding phospholipases (*Pla2g4b, Plag4e,* and *Pla2g5*) and their interacting molecules were altered in KO mice, suggesting a potential bias of cellular events including regulation of cyclic nucleotide-, PLC/calcium-, and/or GTPase-dependent signal transduction (Fig. 3b). Expression of several gene transcripts (*Pak6, Commd7*, and *Ivd*) was dramatically attenuated, whereas *Ifna4* and *Pla2g4e* mRNA levels were highly increased in the KO animals. These results suggest that amygdala function—particularly control of emotional processing—may be influenced by IP_3_K-A.

### Fear- and anxiety-related behavior in IP_3_K-A KO mice

**Figure 4:**
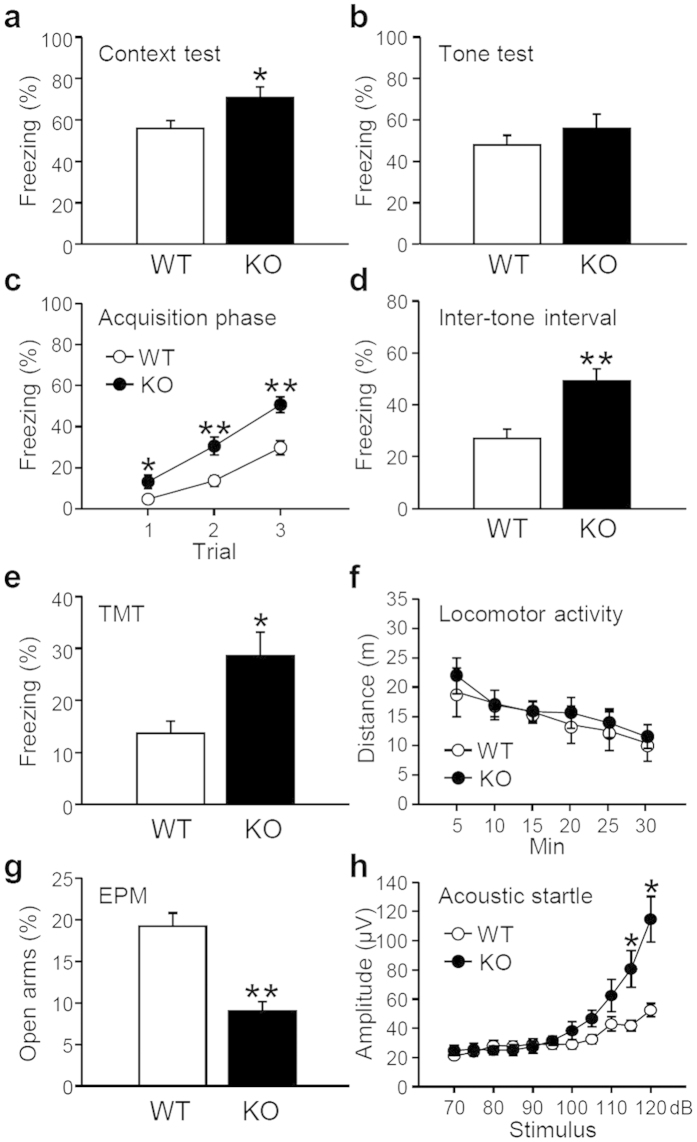
Fear- and anxiety-related behaviors of IP_3_K-A KO mice. (**a**) Freezing percentage at 24 hr after pairing the context and foot shock (WT, n = 14; KO, n = 13). (**b**) The freezing percentage during tone exposure in auditory fear conditioning (WT, n = 14; KO, n = 13). (**c**) Fear memory acquisition curves represented as the percentage of freezing behavior during three repetitions of tone and foot shock paring (WT, n = 14; KO, n = 13; F_(1, 75)_ = 34.15, p < 0.01 for genotype and F_(2, 75)_ = 47.36, p < 0.01 for trial by two-way ANOVA). (**d**) Freezing behaviors during the inter-tone intervals during the training session (WT, n = 14; KO, n = 13). (**e**) Innate fear responses of WT and KO mice after exposure to a synthetic fox feces odor during a 10-min trial (WT, n = 6; KO, n = 5). (**f**) Novelty-induced locomotion in an open field measured for 30 min (WT, n = 6; KO, n = 6). (**g**) Percentage of open arm entries on the elevated plus maze to assess anxiety-related behaviors (WT, n = 7; KO, n = 7). (**h**) Auditory startle reflex elicited by stimuli with different intensities (WT, n = 14; KO, n = 13; F_(1, 275)_ = 27.28, p < 0.01 for genotype and F_(10, 275)_ = 21.20, p < 0.01 for stimulus by two-way ANOVA). All values are mean ± SEM (*p < 0.05 and **p < 0.01 by *t*-test).

The amygdala processes fear information and long-term aversive learning^9^; therefore, we examined whether fear-related learning occurs in IP_3_K-A KO mice. For this purpose, adult male WT and KO mice were tested using contextual and auditory-cued Pavlovian fear conditioning, well-established behavioral paradigms of amygdala-dependent function. During training, the KO and WT mice freely explored the chamber without any differences in freezing behavior, indicating normal motor function in the KO mice. Although impairment in hippocampus-dependent learning were shown in the previous study^7^, IP_3_K-A KO mice had a longer freezing duration under a context-dependent test scheme (Fig. 4a), but showed similar freezing levels in response to conditioned stimuli in the auditory fear conditioning test (Fig. 4b). Interestingly, IP_3_K-A KO mice exhibited a stronger freezing response than WT mice during the acquisition phase of auditory fear conditioning. Stronger freezing responses of the KO mice were found during three successive training sessions (Fig. 4c) as well as the inter-tone intervals (Fig. 4d). It is also noteworthy that the innate fear responses of KO mice were significantly strengthened. Compared with WT mice, they showed more freezing behaviors after exposure to the synthetic predator odor (Fig. 4e). Considering that locomotor activities of KO mice were not significantly impaired in a novel Plexiglas box (Fig. 4f), our findings suggest that IP_3_K-A may affect innate fear-related phenotypes, rather than aversive learning processes or motor activities.

IP_3_K-A KO mice also exhibited anxious phenotypes (Fig. 4g); KO mice spent less time in open arms of the elevated plus maze (EPM) than control mice, indicating a higher state of anxiety. To further assess anxious phenotypes, we carried out the acoustic startle response test, a widely used index of physiological responses to intense and sudden auditory stimuli. It is well known to reflect innate fear and anxiety, although it is also influenced by general sensorimotor functions^16^,^17^. During the test, KO animals displayed a more pronounced startle response to increased auditory stimuli, supporting the idea that IP_3_K-A KO animals have a higher level of innate fear and anxiety (Fig. 4h).

### Electrophysiological properties of IP_3_K-A KO mice

**Figure 5:**
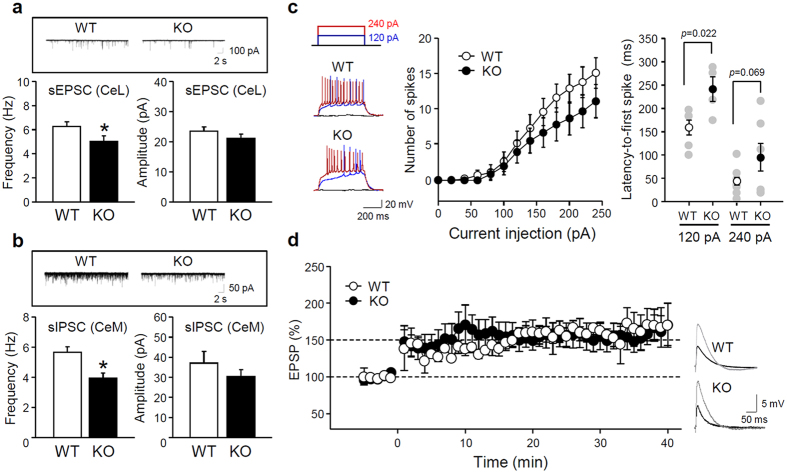
Electrophysiological properties of the amygdala. (**a**) Representative traces and graphs summarizing the spontaneous excitatory postsynaptic currents (sEPSCs) in the CeL (WT, n = 18; KO, n = 14). (**b**) Representative traces and graphs summarizing the spontaneous inhibitory synaptic currents (sIPSCs) in the CeM (WT, n = 9; KO, n = 7; *p < 0.05 by *t*-test). (**c**) The input-output function of neuronal excitability of CeL neurons. Representative traces (left), number of spike (middle, WT, n = 10; KO, n = 7; F_(1, 195)_ = 8.12, p < 0.01 for genotype and F_(12, 195)_ = 18.96, p < 0.01 for current by two-way ANOVA) and latency to the first spike (right, p values by *t*-test). (**d**) High-frequency stimulus-induced EPSP amplitude in thalamo-lateral amygdala (LA) synapses (WT, n = 3; KO, n = 6). All values are mean ± SEM.

Expression of IP_3_K-A in the amygdala—particularly in BLA excitatory neurons and CeA GABAergic interneurons—implies that IP_3_K-A loss can affect neuronal activity in amygdala circuits. As shown in Fig. 5a, sEPSC frequency in the CeL of KO mice was significantly decreased, suggesting that excitatory inputs from LA neurons to CeA inhibitory neurons were weakened. Accordingly, sIPSC frequency in the CeM—an inhibitory output of the amygdala to the brainstem—also was attenuated (Fig. 5b), indicating decreased feed-forward inhibition onto CeA output neurons. We found no significant differences in basic electrophysiological properties between groups, such as resting membrane potential (WT: −66.36 ± 1.51 mV, n = 10; KO: −66.84 ± 1.21 mV, n = 7) and input resistance (WT: 86.86 ± 9.03 MΩ, n = 10; KO: 85.46 ± 8.20 MΩ, n = 7). However, the input-output functions of CeL neurons were significantly attenuated along with a longer latency to the first spike in KO mice (Fig. 5c). We then examined induction and maintenance of LTP in the thalamo-LA pathway, which correlates with auditory fear memory formation. We recorded evoked EPSPs in this pathway and induced LTP by applying high-frequency stimuli (6 trains of 1 sec, 100 Hz stimulation; 1 min inter-train intervals). We found that LTP was not significantly different between WT and KO animals (Fig. 5d), which confirms our finding of normal auditory cue-evoked fear responses in KO mice.

## Discussion

Our study provides the first evidence of functional IP_3_K-A expression in the amygdala and its role in regulating emotion. Genetic deletion of IP_3_K-A altered emotional behavior; the mutant mice had abnormal fear- and anxiety-related behaviors and attenuated synaptic transmission in the CeA. KO mice also exhibited altered expression of multiple amygdala genes, particularly involving metabotropic receptor signaling.

IP_3_K-A was abundantly expressed in multiple amygdaloid nuclei, including the CeA and BLA. Microarray experiments and canonical pathway analysis on DEGs also support the functional importance of IP_3_K-A in the amygdala. IP_3_K-A phosphorylates IP_3_, which is usually produced by multiple PLCs to generate the third-messenger IP_4_ and terminate the initial phase response to the activation of GPCRs and receptor tyrosine kinases (RTKs)^1^. Therefore, it is important that a variety of effector genes controlling calcium homeostasis and signal transduction by cAMP, PLCs, and Rho-GTPases were significantly influenced by IP_3_K-A loss. Concomitant and biased changes in expression of these signaling molecules along with reduced c-Fos expression indicate that IP_3_K-A is required for proper responsiveness of the amygdala to extracellular stimuli.

IP_3_K-A immunoreactivity was found in the GABAergic inhibitory neurons of the CeA and the CAMKIIα-positive excitatory neurons of the BLA. Previous studies only have found IP_3_K-A expressed in excitatory neuronal populations (e.g., hippocampal pyramidal neurons and cerebellar Purkinje cells)^2^. Our findings provide direct evidence that IP_3_K-A plays a role in controlling GABAergic neurons in the CeA. We found decreased c-Fos expression in excitatory and inhibitory neurons and found impaired GABAergic neuron excitability in the CeL of KO mice. Because intracellular increases in IP_3_ are often coupled with increased inhibitory neuron excitability in subdivisions of the CeA^18^, we postulate that the steady-state of intracellular IP_3_ function could be reduced by a feedback mechanism involving a subset of inositol phosphate phosphatases and/or by desensitization of signaling molecules mediating the action of IP_3_.

Our behavioral studies suggest that IP_3_K-A plays a role in controlling emotion. KO mice exhibited a more robust fear response to multiple aversive stimuli and showed more anxious phenotypes than WT mice (Fig. 4). Previous studies reported defects in hippocampal LTP and hippocampus-dependent memory performance using the same mouse model^6^,^7^; therefore, increased freezing behaviors in response to the conditioned context may be due to abnormal brain function related to contextual fear memory formation. However, several lines of evidence collectively support that reinforced innate fear and anxiety, rather than the processing of fear-associated information, can have a larger impact on emotional phenotype. This evidence includes our findings on freezing responses during inter-tone intervals of fear conditioning, freezing responses to predator odor, anxious behaviors on an elevated plus maze, and potentiated acoustic startle responses. Abnormally enhanced innate fear and anxiety are features of anxiety disorders, including PTSD, generalized anxiety disorder, and panic disorder. Our bioinformatics pathway analyses also suggested behavioral alterations related to anxiety, place aversion, and PTSD. Several DEGs in this study (i.e., *Adora2a, Drd2, Pdyn, Gng7,* and *Lcn2)* are associated with onset and severity of anxiety disorders in human genetic studies^19^,^20^ or result in emotional abnormalities in transgenic animal models^21^,^22^,^23^. Interestingly, chronic social-defeat stress leading to depressive and anxious states produces changes in *Adora2a, Drd2, Gng7,* and *Lcn2* mRNA expression in the amygdala^24^. Therefore, genetic deletion IP_3_K-A could disrupt normal amygdala gene expression crucial for controlling emotion, which would result in enhanced fear- and anxiety-related phenotypes.

Our electrophysiological findings also provide evidence of behavioral abnormalities in the KO mice. Recent studies demonstrated that the CeA is an integrative hub for innate fear and anxiety in humans and rodents^25^,^26^. Neural activity in the BLA-CeA circuitry is primarily responsible for autonomic and behavioral control of fear and anxiety. Excitatory inputs from the BLA can activate GABAergic neurons in the CeL, which provide feed-forward inhibition onto inhibitory projection neurons primarily located in the CeM. GABAergic projections from the CeM to hypothalamic nuclei and the periaqueductal gray may mediate autonomic and behavioral fear- and anxiety-related responses^27^. Inhibition of BLA-CeL synapses increases anxiety-related behavior, whereas optogenetic activation of glutamatergic neurons of the BLA has anxiolytic effects^13^. We found that excitatory inputs to the CeL and inhibitory inputs to the CeM were impaired in KO mice (Fig. 5). A disinhibition of afferent pathways from the CeM of KO mice likely underlies their enhanced fear- and anxiety-like responses to aversive stimuli. Multiple GPCR ligands play pivotal roles in integrating neural activity in amygdala circuits and the accompanying emotional states. These ligands include neuropeptides (e.g. cholecystokinin, neuropeptide Y, and corticotrophin-releasing hormone) and neurotransmitters activating their metabotropic receptors^17^,^28^,^29^. Subtle but persistent changes in mediators of GPCR signaling (*Gng7* and regulator of G-protein signaling 2 [*Rgs2*]) can impact anxiety and aggressive behaviors^21^,^30^, and alterations in cAMP, PLC, and Rho GTPase-mediated signaling may cause abnormal neural activity in IP_3_K-A-deficient amygdala circuitry.

In conclusion, this study demonstrates that IP_3_K-A is abundantly and functionally expressed in CeA GABAergic neurons and BLA excitatory neurons. Our molecular and electrophysiological evidence supports our findings of amygdala dysfunction and behavioral abnormalities in reinforced fear and anxiety in IP_3_K-A-deficient mice. Because IP_3_K-A primarily controls intracellular IP_3_ levels and synaptic organization, IP_3_K-A may modulate neuronal excitability mediated by GPCR signaling. Intracellular signaling modulators such as IP_3_K-A are not as commonly discussed for their clinical applications as membrane receptors and their ligands; however, our findings suggest that animal models with altered IP_3_K-A may represent multiple phenotypes of human anxiety disorders. Small molecular modulators of IP_3_Ks recently have been developed, and IP_3_K dysfunction has been implicated in other human diseases such as neurodegenerative disorders, immune disorders, and cancer^31^,^32^. Therefore, clarifying the impact of therapeutically modulating IP_3_K for emotional and cognitive function is critically important.

## Methods

### Animals and tissue preparation

Male IP_3_K-A KO mice and their wild-type (WT) littermate controls from a C57BL/6N background were used at 10 to 15 weeks old. Mice were housed in temperature (22–23 °C)- and humidity (50%)-controlled quarters under a 12-hr light/dark photoperiod (lights on at 08:00a.m.) with free access to food and water. Three-to-five male mice from different litters were housed in a cage after weaning, regardless of their genotype. Then, they were individually housed one week before all experiments. Mice were sacrificed between 02:00p.m. and 04:00p.m. Amygdala tissue was rapidly dissected from 1-mm thick slices and frozen in liquid nitrogen as described previously^33^. All procedures were carried out in accordance with the ethical guidelines of Korea University and with the approval of the Animal Care and Use Committee of Korea University.

### Immunohistochemistry

Immunohistochemistry was performed as described previously^7^. Briefly, mice were perfused transcardially with 0.9% NaCl containing 25 U/mL heparin, then 4% paraformaldehyde in tris-buffered saline (pH 7.4). Brains were removed, post-fixed overnight at 4 °C in the same fixative, and cryoprotected in a series of solutions (10%, 20%, and 30% sucrose in phosphate-buffered saline [PBS]). Frozen brains were coronally cut (40 μm thick). Sections were blocked with PBS containing 5% normal goat serum and 0.2% Triton X-100 for 30 min and incubated with anti-IP_3_K-A (Santa Cruz Biotechnology, Dallas, TX), anti-NeuN, anti-GFAP, anti-CamKIIα (Invitrogen, Carlsbad, CA), anti-GAD67 (Millipore, Billerica, MA), or anti-c-Fos (Santa Cruz Biotechnology) antibodies overnight at 4 °C. After PBS washes, sections were incubated with Cy3, Cy5, or Alexa Fluor^®^ 488-conjugated secondary antibodies (Jackson ImmunoResearch Laboratories, West Grove, PA) for 30 min at room temperature. After three washes, sections were mounted with aqueous mounting medium (Biomeda, Foster City, CA) and observed on an Axiovert 200 Fluorescent microscope (Zeiss, Oberkochen, Germany).

### Microarray analysis

Analysis of RNA using microarray was performed as previously described^34^ with minor modifications. Total RNA was extracted using RNeasy Mini kits (Qiagen, Valencia, CA) and quality was evaluated with an Agilent 2100 Bioanalyzer (Agilent, Santa Clara, CA). Three sets of pooled samples (i.e., from 6 to 8 mice per genotype from three litters) were independently prepared and analyzed. For each sample, 200 ng RNA was amplified, labeled, and analyzed using GeneChip Mouse Gene 2.0 ST arrays (Affymetrix, Santa Clara, CA) according to manufacturer’s protocols. The data from microarray analyses were deposited in the Gene Expression Omnibus (GEO) database (Accession #: GSE77001). Differentially expressed genes (DEGs; more than 1.2-fold changes at p < 0.1 by Student t’s test) were used to construct a dataset and analyzed using the “core analysis” function of Ingenuity Pathway Analysis^TM^ (IPA; www.ingenuity.com; Qiagen). We used “canonical pathway analysis” and “biological functions/disease annotation” to explore affected pathways and predict alterations in amygdala function.

### Quantitative Reverse Transcription-Polymerase Chain Reaction (qRT-PCR)

For DEGs identified in the microarray analysis, we validated alterations in expression using qRT-PCR as described previously^34^. We reverse-transcribed 500 ng RNA with MMLV reverse transcriptase (Promega, Fitchburg, WI). cDNA underwent qPCR with SYBR Green I and ROX as a passive dye (Sigma Aldrich, St. Louis, MO). TATA box-binding protein was the internal control. Primer sequences are listed in Table S1.

### Behavioral studies

Behavioral assessments were performed as described previously with modifications^33^,^35^. Mice were not repeatedly used for multiple behavioral experiments.

#### Fear conditioning

The square type-training chamber (20 × 15 × 25 cm) had four transparent walls and a foot-shocking grid floor; here, training and context tests were performed. The tone test was performed in a 20-cm diameter × 30-cm height transparent cylindrical cage with bedding. All mice were habituated to the conditioning chamber for 2 days (10 min/day). Conditioning began with presentation of the tone (conditioned stimulus [CS]; 30 sec at 80 dB/3000 Hz) with the foot shock (unconditioned stimulus; 0.4 mA) delivered during the last 2 sec of CS presentation. The intertrial interval lasted 30 sec. Context tests occurred 24 hr after conditioning, and tone tests occurred 48 hr after conditioning. Context memory tests occurred in the training chamber without CS for 3 min, and tone memory was measured in the transparent cylindrical cage using the same procedure but without electric shocks. During each trial, freezing (indicating fear memory) was measured by an automatic system (SmartEye^®^, Göthenburg, Sweden).

#### Novelty-induced locomotion

Mice were individually placed into a novel Plexiglas box (40 × 40 × 30 cm) for 30 min. Locomotor activities were recorded by an overhead video camera connected to a computer with tracking software (Ethovision 3.1, Noldus, Leesburg, VA).

#### Innate fear response to predator odor

To examine innate fear, mice were placed in a chamber with a beaker containing 30 μl synthetic fox feces odor trimethylthiazoline (TMT, Contech Enterprises Inc., Victoria, Canada) as previously reported. TMT-evoked freezing behavior was recorded for 10 min after TMT exposure and scored as average percentage of freezing time in the last 4 min.

#### Elevated plus maze

Consisting of two open and two enclosed arms (5 × 30 cm) with 20 cm-high walls, the apparatus was elevated 50 cm. Mice were individually placed in the center facing an open arm and allowed to explore for 10 min. Frequency and duration of open- and closed-arm entries were recorded. Entry was defined as movement of all paws into an arm. The percentage of time in the open arms was scored.

#### Acoustic startle test

Acoustic startle measure is based on reflexive whole-body flinches (startle responses) following exposure to a sudden noise, as described previously^36^. Startle responses to acoustic stimuli were determined using a standard startle chamber, a soundproof apparatus with a plexiglass cylinder to hold animals and a speaker producing sound bursts. Animal motion was detected via a load cell transducer below the cylinder. Prior to testing, animals were acclimatized to the chamber for 30 min for 3 days with background white noise at 60 dB. After 5 min of habituation, mice underwent five sessions of random sound bursts from 70 dB to 120 dB in 5 dB intervals (55 trials total, 40 ms duration, 60 sec intertrial interval).

### Electrophysiology

#### Slice preparation

Isolated whole brains were placed in ice-cold artificial cerebrospinal fluid (aCSF) containing (in mM): 175 sucrose, 20 NaCl, 3.5 KCl, 1.25 NaH_2_PO_4_, 26 NaHCO_3_, 1.3 MgCl_2_, and 11 D-(+)-glucose and gassed with 95% O_2_/5% CO_2_. Coronal slices (300 μm) were cut with a vibroslicer (NVSL, World Precision Instruments, Sarasota, FL) and incubated in aCSF containing (in mM): 120 NaCl, 3.5 KCl, 1.25 NaH_2_PO_4_, 26 NaHCO_3_, 1.3 MgCl_2_, 2 CaCl_2_, and 11 D-(+)-glucose and gassed with 95% O_2_/5% CO_2_.

#### Whole-cell patch-clamp recordings

Recordings were made using an EPC10 amplifier (HEKA Elektronik, Lambrecht, Germany). Signals were sampled at 5 kHz and filtered at 3 kHz with the Bessel filter of the amplifier. We used pipettes (resistance, 2.5–3.5 MΩ) filled with (in mM): 120 KCl (for spontaneous inhibitory postsynaptic currents [sIPSCs]) or K-gluconate (for intrinsic properties and spontaneous excitatory postsynaptic currents [sEPSCs] at the central amygdala or evoked excitatory postsynaptic potentials [eEPSPs] at the thalamo-lateral amygdala [LA]), 10 HEPES, 1 MgCl_2_, 5 NaCl, 0.2 EGTA, 3 QX314, 2 Mg-ATP, and 0.3 Na-GTP (pH 7.2 using KOH; osmolarity ~297 mmol/kg using sucrose). Neurons were voltage-clamped at –50 mV for sIPSC or −70 mV for sEPSC. Solutions were perfused via superfusion by gravity at 1.3 to 1.5 mL/min and maintained at 32 ± 1 °C. After whole-cell configuration, series resistance was regularly monitored, and if changed by >20%, the data were discarded. sEPSCs and sIPSCs were analyzed using MiniAnalysis software (Synaptosoft, Decatur, GA). The detection threshold was three times the RMS noise, and data were discarded if the rise time was higher than 4 ms and the decay time was greater than 10 ms. RMS noise of WT and KO mice were not different (medial CeA [CeM] sIPSC: WT, n = 9, 2.73 ± 0.20 pA; KO, n = 7, 3.07 ± 0.23 pA; unpaired t-test, p = 0.2719; lateral CeA [CeL] sEPSC: WT, n = 18, 3.02 ± 0.24 pA; KO, n = 14, 2.94 ± 0.20 pA; unpaired t-test, p = 0.7981). Peaks were selected from the part recorded at least 10 min after treatment with NBQX (10 μM) and D-APV (30 μM) for sIPSCs or picrotoxin (100 μM) for sEPSCs. Consequence 300 peaks were collected from detected peaks for further analysis. To record evoked EPSPs at the thalamo-LA pathway, the stimulation electrode was placed at the midpoint of the trunk between the internal capsule and the medial boundary of the LA. Thalamic afferents were stimulated at 0.067 Hz using a concentric bipolar electrode. The baseline amplitudes of the evoked EPSPs were adjusted to 4 to 7 mV. LTP was induced with 6 trains of 100 stimuli at 100 Hz (15-sec interval) with 100 μM picrotoxin to inhibit GABAergic inputs.

### Statistical analysis

Group differences were evaluated using Student’s t-test or two-way analyses of variance (ANOVA) followed by Newman-Keuls comparisons. p < 0.05 was considered significant. All statistics are mean ± SEM.

## Additional Information

**How to cite this article**: Chung, S. *et al*. The role of inositol 1,4,5-trisphosphate 3-kinase A in regulating emotional behavior and amygdala function. *Sci. Rep.* **6**, 23757; doi: 10.1038/srep23757 (2016).

## Supplementary Material

Supplementary Information
