# Supplementary material for: The role of inositol 1,4,5-trisphosphate 3-kinase A in regulating emotional behavior and amygdala function
Source: Sci Rep. 2016 Apr 7;6:23757. doi: 10.1038/srep23757 (PMC4823716; doi:10.1038/srep23757)
Supplement: Supplementary Information [file srep23757-s1.pdf]

**Supplementary information to:**

# The role of inositol 1,4,5-trisphosphate 3-kinase A in regulating emotional behavior and amygdala function

Sooyoung Chung[1,2,8], Il Hwan Kim[3,8], Dongmin Lee[4,8], Kyungjoon Park[5], Joo Yeon Kim[1,4], Yeon Kyung Lee[6], Eun Joo Kim[7], Hyunwoo Lee[1,4], June-seek Choi[6], Gi Hoon Son[1], Woong Sun[1,4], Kisoon Shin[6], Hyun Kim[1,4*]

**Table S1. Primer sequences of target genes for quantitative RT-PCR analysis**

| Gene | Accession No. | Forward (5'→3') | Reverse (5'→3') |
|---|---|---|---|
| Adora2a | NM_009630 | GTCCTGGTCCTCACGCAGAGTTCCATC | AGCCATTGTACCGGAGTGGA |
| Drd2 | NM_010077 | TGCCATTGTTCTTGGTGTGT | GTGAAGGCGCTGTAGAGGAC |
| Pdyn | NM_018863 | CTGGACAGGAGAGGAAGCAG | TGTGTGGAGAGGGACACTCA |
| Gng7 | NM_001038655 | ATCTCTGCCCACACTTCCAG | ATCAGCCAAGCACGTCCTAC |
| Lcn2 | NM_008491 | TGGAAGAACCAAGGAGCTGT | CACACTCACCACCCATTCAG |
| c-Fos | NM_010234 | AGTCAAGGCCTGGTCTGTGT | TCCAGCACCAGGTTAATTCC |
| Pla2g4b | NM_145378 | AACAGTTTAGGCCGGAGTGA | CTTGAACTCGGAGCAGAAGG |
| Pla2g4e | NM_177845 | TAAAAGCCAAAGGGCAGCTA | CAGGACTGTGGAGGGATCAT |
| Pla2g5 | NM_001122954 | AAGCCAGACCTGGGGTTTAT | GAGCTGATTGTTGCCCTCTC |
| Btk | NM_013482 | CGAGATAAAAATGGGCAGGA | CTTCGAGTCATGTGCTTGGA |
| Pak6 | NM_001033254 | CCTAAGGACATGGAAGGCA | TCCCAGAACCTTCAGAGGAC |
| Myl12b | NM_023402 | ACGGGGGACAATGTTGTAAA | TCCAGCAAAACCAGAAATCC |
| Chp1 | NM_019769 | AGGCTGCTTCTGTCCTGTGT | GATAGCCTTCCAAAGCAACG |
| Ppp1cb | NM_172707 | ATTACCCTCACCTGCATTGG | TCCAAGGCTTGTCAAAAAGG |
| Serpina5 | NM_172953 | GCCAAGAAGCAGATCAACAA | ATCATGACATGGGTGCTGTC |
| Commd7 | NM_133850 | TAGGGCCTTGTGGATTCAAG | GGGGTCCTCACGTCTACTCA |
| Ifna4 | NM_010504 | GATGTGACCTGCCTCACACT | TTATCCACCTTCTCCAAGGG |
| Ivd | NM_019826 | CAGGAAGGACAGAGTCCACA | AGCCTGTGACAGAGTGATGC |
| Tbp | NM_013684 | GGGAGAATCATGGACCAGAA | CCGTAAGGCATCATTGGACT |
